# Yeast Assay Highlights the Intrinsic Genomic Instability of Human PML Intron 6 over Intron 3 and the Role of Replication Fork Proteins

**DOI:** 10.1371/journal.pone.0129222

**Published:** 2015-06-08

**Authors:** Roland Chanet, Guy Kienda, Amélie Heneman-Masurel, Laurence Vernis, Bruno Cassinat, Philippe Guardiola, Pierre Fenaux, Christine Chomienne, Meng-Er Huang

**Affiliations:** 1 UMR3348 “Genotoxic Stress and Cancer”, Centre National de la Recherche Scientifique, Orsay, France; 2 Institut Curie, Centre de Recherche, Orsay, France; 3 AP-HP, Hôpital Saint-Louis, Paris, France; 4 Inserm UMRS-1131, Hôpital Saint-Louis, Université Paris Diderot, Paris, France; 5 Plateforme SNP, Transcriptome & Epigénomique, Centre Hospitalier Universitaire, Angers, France; RIKEN Advanced Science Institute, JAPAN

## Abstract

Human acute promyelocytic leukemia (APL) is characterized by a specific balanced translocation t(15;17)(q22;q21) involving the PML and RARA genes. In both *de novo* and therapy-related APL, the most frequent PML breakpoints are located within intron 6, and less frequently in intron 3; the precise mechanisms by which these breakpoints arise and preferentially in PML intron 6 remain unsolved. To investigate the intrinsic properties of the PML intron sequences *in vivo*, we designed *Saccharomyces cerevisiae* strains containing human PML intron 6 or intron 3 sequences inserted in yeast chromosome V and measured gross chromosomal rearrangements (GCR). This approach provided evidence that intron 6 had a superior instability over intron 3 due to an intrinsic property of the sequence and identified the 3’ end of intron 6 as the most susceptible to break. Using yeast strains invalidated for genes that control DNA replication, we show that this differential instability depended at least upon Rrm3, a DNA helicase, and Mrc1, the human claspin homolog. GCR induction by hydrogen peroxide, a general genotoxic agent, was also dependent on genetic context. We conclude that: 1) this yeast system provides an alternative approach to study in detail the properties of human sequences in a genetically controlled situation and 2) the different susceptibility to produce DNA breaks in intron 6 versus intron 3 of the human PML gene is likely due to an intrinsic property of the sequence and is under replication fork genetic control.

## Introduction

Chromosome translocations and the resulting fusion proteins are closely associated with leukemogenesis. The balanced translocation t(15;17)(q22;q21) involving the PML and RARA genes generates PML-RARA fusion protein, a determinant factor triggering acute promyelocytic leukemia (APL) [1,2]. The genomic breakpoints in the RARA gene on chromosome 17 localize exclusively within intron 2, while those in the PML gene on chromosome 15 have been positioned either within the intron 3 (bcr3), exon 6 (bcr2) or intron 6 (bcr1). Interestingly, the majority (2/3) of the breakpoints involving the PML gene are localized in the intron 6 [3,4]. This preference of intron 6 versus intron 3 is also found in therapy-related secondary APL (t-APL) [4]. Breakpoint analysis on a limited number of secondary or therapy-induced APL (t-APL) cases reveals the presence of an 8-base pair “hotspot” region in PML intron 6 in patients receiving mitoxantrone, while the site preferences for epirubicin-induced APL is less clear-cut but different from the mitoxantrone-associated hotspot [5–7]. Functional *in vitro* assay indicates that these breakpoint sequences are preferential sites for mitoxantrone-induced or epirubicin-induced DNA topoisomerase II cleavage.

No study has assessed whether the higher frequency of PML-RARA bcr1, involving the PML intron 6 in both *de novo* and t-APL, or the hotspots on intron 6 identified in t-APL, result from intrinsic sequence-specific properties *per se*, or from some selected random events that lead to leukemia. The use of the yeast *Saccharomyces cerevisiae*, with its available genetic assays and facility for genetic manipulation, provides a powerful *in vivo* approach to obtain relevant insights. *S*. *cerevisiae* has been used as model organism to decipher metabolic pathways controlling stability of human repetitive sequences such as Alu repeats, trinucleotide repeats, and mini- or micro-satellites [8–12]. These studies provided detailed insights in the mechanisms involved in these processes. A yeast-based assay has also been developed to study the first steps of gross chromosomal rearrangements (GCR), *i*.*e*. the DNA break followed by either telomere addition or non-reciprocal translocation [13,14], thus overcoming the problems associated with a further selection in order to generate a functional product. This assay, called GCR assay, allows to perform detailed analysis of the genetic control of chromosomal rearrangements [15].

In the present study, we used the GCR assay to study the molecular mechanisms underlying the instability of the human PML gene introns implicated in the t(15;17) translocation. PML intron 3 and intron 6 sequences were inserted in the yeast chromosome V region between *CIN8* and *NPR2* genes, a region known to be free of any particular instability in GCR assay [16], for a detailed comparative study. We observed that intron 6 is indeed significantly less stable compared to intron 3 in the yeast assay. Using yeast mutants we show for the first time that this differential instability is genetically controlled.

## Materials and Methods

### Media and strains

*S*. *cerevisiae* strains were grown in standard media including yeast extract peptone dextrose medium (YPD) or synthetic complete medium lacking appropriate amino acids as indicated. Canavanine- and 5-fluoroorotic acid (5FOA)-resistant mutants (Can^R^-5FOA^R^) resulted from the loss of the region including *CAN1* and *URA3* on chromosome V were selected on synthetic complete medium lacking arginine and uracil but containing 60 mg/L of canavanine and 1 g/L of 5FOA. Hydrogen peroxide (H_2_O_2_, Sigma-Aldrich) was added at 1 mM final concentration.

**Fig 1:**
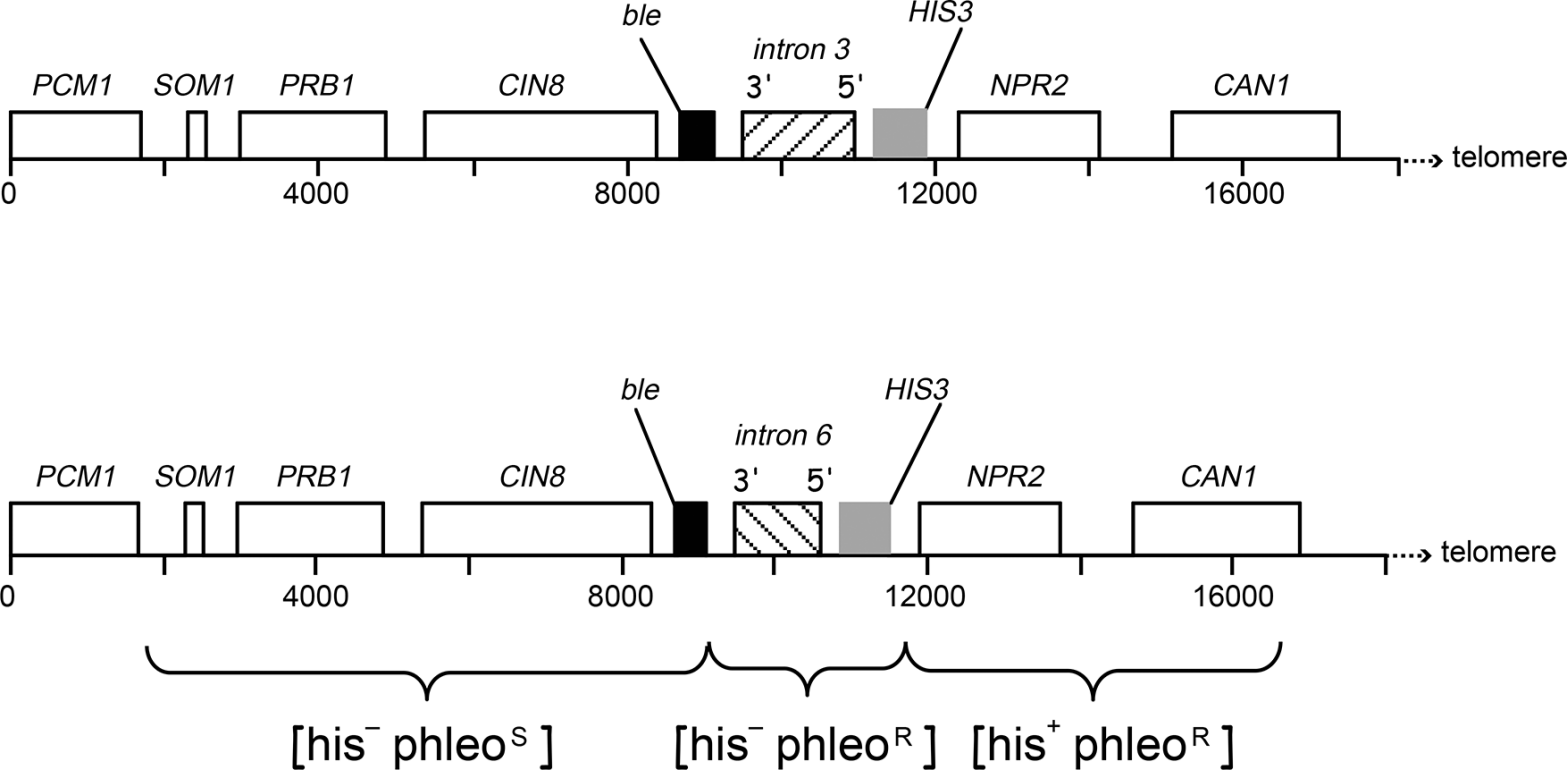
Structure of left end of chromosome V of RDKY3615 strain in which intron cassettes were introduced between *CIN8* and *NPR2* genes. The yeast genes are approximately drawn to scale. The *URA3* gene located 7.5 kb telomeric to *CAN1* is not shown. All DNA breaks that occur in the window between *PCM1* and *CAN1* gene lead to loss of *CAN1-URA3* region, resulting in the production Can^R^-5FOA^R^ cells. The *ble* and *HIS3* selectable markers flanking introns confer resistance to phleomycin and growth in the absence of histidine, allowing the GCR mutants [Can^R^-5FOA^R^] to be classified into three phenotypic categories: [his^−^phleo^S^], [his^−^phleo^R^] and [his^+^ phleo^R^].

The strains used in this study for the analysis of chromosome rearrangements were all isogenic to the strain RDKY3615 (*MATa*, *ura3-52*, *leu2∆1*, *trp1∆63*, *his3∆200*, *lys2∆Bgl*, *hom3-10*, *ade2∆1*, *ade8*, *hxt13*::*URA3*) [14] (Table 1). Gene replacements were made by standard PCR-based homology-directed methods. The resulting construction of PML intron 3- or intron 6-containing strains is illustrated schematically in Fig 1. First, a fragment containing the *HIS3* genes flanked by sequences homologous to the upstream and downstream sequences of yeast chromosomal target site, obtained by PCR amplifying the *HIS3* gene present on plasmid pRS303 using primers 663 and 664 (S1 Table), was introduced into the RDKY3615 strain. The correct integration at the 3’ end of *NPR2* gene was confirmed by PCR, resulting in strain RC2501. Second, PML intron 3 (1443 bp) and intron 6 (1063 bp) were amplified from human genomic DNA with pairs of primers 665/666 and 667/668 respectively, that contain the restriction sites for HindIII and BglII. The HindIII- and BglII-digested PCR fragments, 1471 and 1090 bp respectively, were then inserted into the HindIII- and BglII-cut pUG66 plasmid [17], next to the *ble* marker that confers phleomycin resistance [phleo^R^]. Third, fragments containing intron 3 and *ble*, or intron 6 and *ble*, flanked by sequences homologous to the upstream and downstream sequence of yeast chromosomal targeted site, were generated by PCR using pUG66-intron 3 or pUG66-intron 6 as a template and 668/670 or 670/671 primer pair. These fragments were used to transform RC2501 strain to integrate into the chromosomal site next to *HIS3*, yielding the strains RC2540 (containing intron 3) and RC2553 (containing intron 6). The correct integration of cassettes *ble-intron 3-HIS3* and *ble-intron 6-HIS3* between *CIN8* and *NPR2* genes of original RDKY3615 strain was confirmed by genomic PCR. Deletion mutants *pol32*::*TRP1*, *rrm3*::*KanMX*, *elg1*::*KanMX and mrc1*::*TRP1* were constructed by direct gene replacements or by classical genetics through crosses with isogenic counterparts followed by tetrad dissection. All the primers used in strain construction are listed in S1 Table.

**Table 1:** Yeast strains used in this study.

Strain	Genotype
RDKY3615	*MAT*a *ura3-52 leu2∆ trp1∆63 his3∆200 lys32∆Bgl hom3-10 ade2∆1 ade8 yel069*::*URA3*
RC2540	*MAT*a *ura3-52 leu2∆ trp1∆63 his3∆200 lys32∆Bgl hom3-10 ade2∆1 ade8 HIS3 intron 3 ble yel069*::*URA3*
RC2553	*MAT*a *ura3-52 leu2∆ trp1∆63 his3∆200 lys32∆Bgl hom3-10 ade2∆1 ade8 HIS3 intron 6 ble yel069*::*URA3*
RC2626	same as RC2540 *pol32*::*TRP1*
RC2620	same as RC2553 *pol32*::*TRP1*
RC2689	same as RC2540 *rrm3*::*KanMX*
RC2690	same as RC2553 *rrm3*::*KanMX*
RC2638	same as RC2540 *elg1*::*KanMX*
RC2632	same as RC2553 *elg1*::*KanMX*
RC2614	same as RC2540 *mrc1*::*TRP1*
RC2609	same as RC2553 *mrc1*::*TRP1*

The strains RC2540 and RC2553 are derived from strain RDKY3615 and are all isogenic except for the indicated markers.

### Measurement of GCR rates

The rate of accumulation of GCR was determined by fluctuation analysis. For each experiment, a fresh yeast colony was suspended in 500 mL YPD, grown to reach ~1 × 10^5^ cells/mL, and distributed to 15 parallel cultures, each with 10 mL. For induced GCR formation, H_2_O_2_ was added to the culture at this stage at 1 mM final concentration. These parallel cultures were further grown at 30°C up to stationary phase, harvested, washed and suspended in sterile water. Appropriate dilutions for each culture were plated on YPD plates for total cell counts and remaining cells on selection plates containing canavanine and 5FOA to identify GCR mutants. Colonies were counted after four to five days of growth at 30°C. For each strain, GCR measurement was repeated for at least three times for a total of at least 45 independent cultures. GCR rate with 95% confidence intervals was calculated according to Hall *et al*. [18], using the MSS Maximum Likelihood Method available at the keshavsingh.org website. To classify the GCR events (Can^R^-5FOA^R^) from each culture into three categories, [his^−^phleo^S^], [his^−^phleo^R^] and [his^+^ phleo^R^], Can^R^-5FOA^R^ colonies from each culture were streaked on selection plates containing canavanine and 5FOA and further replica plated on complete medium lacking histidine or containing phleomycin (40 μg/mL). The number of each type of clones in each culture was estimated and the rate of each category of GCR was calculated by using the same method as for total GCR rate [18].

### Mapping of rearrangement breakpoints by PCR

Rearrangement breakpoint positions in independent GCR clones harboring [his^−^phleo^R^] phenotype were mapped by PCR analysis of yeast genomic DNA using primer pairs covering *ble* through *HIS3* chromosomal region; this located a breakpoint to the zone in the telomeric direction that did not yield a PCR product. In the 3’ region of the two introns, the breakpoints were further refined by PCR analysis of the relevant zone using 4–5 pairs of additional PCR primers that amplified overlapping segments that were progressively shifted by ~50 bp. By analysis of the primer pairs that did and did not amplify fragments from the mutant genomic DNA, it was possible to locate a breakpoint to ~50 bp. The primer pair that amplifies a portion of the *PCM1* essential gene was included in each sample to verify the effectiveness of the PCR reaction. The sequences of independent rearrangement breakpoints were determined following procedures previously described [14]. All primers used in mapping and sequencing of GCR breakpoints are available upon request.

## Results

### Intron 6 displays higher spontaneous instability than intron 3

In order to study the intrinsic properties of the intron 3 and intron 6 sequences of the PML gene, we constructed strains where each intron sequence bracketed between two selectable markers, *ble* and *HIS3*, was inserted between the *CIN8* and *NPR2* genes of the yeast chromosome V, a region where the probability of DNA break is constant [16,19]. The *ble* and *HIS3* selectable markers confer resistance to phleomycin and growth in the absence of histidine (Fig 1). The GCR mutants [Can^R^-5FOA^R^] could thus be classified into three phenotypic categories through simple replica plating: 1) the breaks occurring between the first essential gene (*PCM1*) and the 3’ end of inserted *ble* gene (7.4 kb interval, designated pcm-ble region) confer a [his^−^phleo^S^] phenotype; 2) the breaks involving the introns (2.1 kb and 1.7 kb for intron 3 and intron 6 respectively, designated intron region) give rise to a [his^−^phleo^R^] phenotype; 3) and finally, the breaks occurring between the inserted *HIS3* gene and the *CAN1* gene (4.9 kb, designated his-can region) result in a [his^+^ phleo^R^] phenotype. Fluctuation analysis showed that the spontaneous GCR rate of wild-type intron 6-containing strain was 16.8 × 10^−10^, significantly higher than that of the intron 3-containing strain which was 2.7 × 10^−10^ (Table 2). It is to note that the GCR rate of the intron 3-containing strain was similar to that of the RDKY3615 strain containing no intron (2.6 × 10^−10^), and was closed to the reported GCR rate of the same strain (3.5 × 10^−10^) [16]. These data indicate that the presence of intron 3 has no effect on the stability of the chromosomal region covering *PCM1-CAN1*, while the insertion of intron 6 sequence significantly increased the GCR rate.

**Table 2:** Spontaneous and induced GCR rates of intron 6- or intron 3-containing strains.

Strain		Total GCR rate	[his^+^ phleo^R^]	[his^−^phleo^R^]	[his^−^phleo^S^]
Intron 6, WT	spontaneous	16.8 (11.0–23.3)	1.8 (0.7–2.9)	11.6 (7.2–16.6)	2.7 (1.2–4.6)
	H2O2	35.7 (25.7–47.2)	3.9 (1.9–6.5)	17.5 (11.5–24.5)	9.8 (5.8–14.6)
Intron 6, *pol32*	spontaneous	43.8 (33.3–55.2)	5.4 (3.0–8.1)	22.5 (16.0–29.8)	2.3 (1.0–3.8)
	H2O2	43.5 (32.4–55.8)	3.3 (1.6–5.5)	19.6 (13.2–26.7)	7.2 (4.2–10.9)
Intron 6, *rrm3*	spontaneous	3.5 (2.3–4.9)	0.4 (0.2–0.7)	2.3 (1.4–3.4)	0.6 (0.3–1.0)
	H2O2	20.2 (14.2–26.8)	5.7 (3.3–8.5)	13.2 (8.8–18.2)	3.9 (2.0–6.0)
Intron 6, *elg1*	spontaneous	83.4 (67.4–100.5)	10.0 (6.4–14.0)	48.0 (37.2–60.0)	16.0 (11.0–21.7)
	H2O2	185.4 (153.4–219.5)	12.8 (8.0–18.5)	84.2 (65.8–104.3)	31.2 (22.0–41.4)
Intron 6, *mrc1*	spontaneous	30.5 (22.1–39.9)	9.3 (5.7–13.5)	6.6 (3.8–10.0)	6.3 (3.6–9.6)
	H2O2	26.2 (18.0–35.4)	8.0 (4.5–12.1)	10.5 (6.3–15.4)	6.7 (3.8–10.6)
Intron 3, WT	spontaneous	2.7 (1.2–4.6)	2.1 (0.9–3.7)	1.3 (0.4–2.5)	0.9 (0.2–1.8)
	H2O2	21.5 (14.5–29.5)	7.0 (3.0–10.0)	5.2 (2.2–8.2)	7.5 (4.5–11.5)
Intron 3, *pol32*	spontaneous	12.9 (8.4–18.2)	1.6 (0.6–3.0)	7.4 (4.4–11.0)	2.6 (1.2–4.4)
	H2O2	16.8 (10.6–24)	7.2 (3.9–11.4)	5.6 (2.8–9.0)	3.9 (1.8–6.7)
Intron 3, *rrm3*	spontaneous	5.7 (3.4–8.4)	1.4 (0.6–2.4)	2.7 (1.4–4.2)	2.6 (1.3–4.1)
	H2O2	25.8 (18.4–34.0)	11.8 (7.6–16.7)	11.6 (7.4–16.4)	6.1 (3.5–9.3)
Intron 3, *elg1*	spontaneous	38.3 (29.0–48.5)	5.6 (3.3–8.4)	8.9 (5.6–12.7)	17.0 (11.8–22.9)
	H2O2	103.3 (83.0–126.0)	13.1 (8.3–18.7)	41.5 (30.6–53.5)	29.2 (20.7–38.7)
Intron 3, *mrc1*	spontaneous	26.2 (18.3–35.1)	8.2 (4.8–12.3)	4.0 (2.0–6.5)	9.6 (5.7–14.1)
	H2O2	25.2 (17.0–34.4)	3.9 (1.8–6.5)	7.9 (4.4–12.2)	8.3 (4.7–12.6)

GCR rate is expressed as × 10^−10^ GCR events per generation calculated according to MSS Maximum Likelihood Method [18]. The numbers in parenthesis refer to the 95% confidence interval. For the definition of all phenotypic categories, see text. GCR rate for RDKY3615 strain determined under same conditions is 2.6 (0.7–5.3) × 10^−10^.

We then classified the GCR mutants (Can^R^-5FOA^R^) from each culture into three categories, [his^−^phleo^S^], [his^−^phleo^R^] and [his^+^ phleo^R^], through simple replica plating on complete medium lacking histidine or containing phleomycin. The number of each type of clones in each culture was estimated and the rate of each category of GCR event was calculated. As shown in Table 2, the intron 6 region was significantly more instable than the region containing intron 3. In addition, as the length of intron 6 is 73% of intron 3, the instability of intron 6 per “unit of length” is more than 10 times greater than that of intron 3.

As the introns are surrounded by two “spacer” sequences, 390 bp between *ble* gene and intron, and 275 bp between intron and the *HIS3* gene, we determined by PCR in the [his^−^phleo^R^] GCR clones that the DNA breaks did occur in the intron and not in the flanking “spacer” sequences. Roughly 6% of the breaks occurred in the 3’ flanking sequence and 10% in the 5’ flanking sequence of the intron 6, and 16% and 30% in the respective flanking sequence of the intron 3. We noted also that the GCR rates involving pcm-ble or his-can regions were not statistically different between the two strains. Thus, the GCR rate of [his^−^phleo^R^] events truly reflects the intrinsic susceptibility of the intron 6. Taken together, we conclude that, in the same chromosomal environment, the intron 6 sequence of the human PML gene is more susceptible to spontaneous breaks than intron 3.

### Effects of H_2_O_2_ treatment on intron breakpoints

PML gene breakpoints in human t-APL induced by anti-topoisomerase II drugs involve more frequently intron 6 than intron 3 [4,7]. We tried to study whether the same type of bias could be found when intron 6- and intron 3-containing wild-type strains were treated with epirubicin at 10 μg/mL, a concentration that does not inhibit yeast cell growth. Treatments with epirubicin at this low concentration did not significantly increase the global GCR rate of the intron 6-containing wild-type strain (data not shown), very probably due to the presence of efficient multi-drug resistance pump system [20,21]. Although several mutants specifically sensitive to anti-topoisomerase have been reported [22], though not characterized, the effects of anti-topoisomerases on intron 6 and intron 3 in our yeast assay were not further studied as the genetic background of the strains used for these studies were different from that used for GCR detection

We then tested whether a general genotoxic agent that should not be pumped out by multi-drug resistance pump system can have impact on the stability of intron 6 and intron 3 in GCR assay. We treated the intron 6- and intron 3-containing strains with 1 mM H_2_O_2_, a concentration that does not inhibit growth. The number of GCR events was globally increased in the presence of H_2_O_2_ compared to untreated cultures. Intron 6-containing strain displayed higher GCR rate (35.7 × 10^−10^) than intron 3-containing strain (21.5 × 10^−10^) in the presence of H_2_O_2_ (Table 2). Interestingly, the increased GCR events in both intron-containing strains were mainly seen in the larger pcm-ble region that covers 7.4 kb, and proportionally less in the his-can region (4.9 kb) and the intron region. This observation could suggest that H_2_O_2_-induced DNA damage favors the GCR formation scattered on these three sub-regions regardless of the intron sequence. However, when the size of each region was taken into account, the instability of intron 6 or intron 3 per “unit of length” following H_2_O_2_ treatment was significantly higher compared to flanking pcm-ble and his-can regions, and the instability of intron 6 per “unit of length” following H_2_O_2_ treatment was significantly higher than intron 3. Therefore, intron 6 sequence appears to be more susceptible to DNA damage and genomic instability induced by a general genotoxic agent H_2_O_2_.

### Susceptibility of intron 6 to spontaneous and H_2_O_2_-induced DNA breaks is under genetic control

Having shown that in wild-type context, the intron 6 of PML gene is more susceptible to DNA breaks than the intron 3, either spontaneously or after H_2_O_2_ exposure, we raised the question whether this increased susceptibility was genetically controlled. We chose to study the impact of 4 candidate genes of interest, *POL32*, *RRM3*, *ELG1* and *MRC1*, due to their involvement in controlling genomic stability during DNA replication [23]. Pol32 is a structural component of the lagging strand DNA polymerase δ as well as the mutagenic polymerase ζ [24]; *pol32∆* mutants are also deficient in Break Induced Replication [25]. Rrm3 is a DNA helicase having functions in maintaining genome stability during replication [26,27]. Elg1 is a well-conserved protein that may be involved in unloading PCNA clamp in order to prevent replication fork stalling [28,29]. The persistence of modified PCNA and associated proteins after DNA replication in the absence of Elg1 was reported to lead to DNA breaks that give rise to high genomic instability [30]. Finally, Mrc1, the yeast homolog of human claspin, is involved in checkpoint control with the leading strand DNA polymerase ε [31,32].

In a *pol32∆* context, as expected [33], the spontaneous global GCR rates were increased by 2 to 5 folds in either intron 6 or intron 3-containing strains compared to wild-type *POL32* strain. Consequently, the difference in stability between the two types of introns is maintained (Table 2). The increased GCR rate in *pol32∆* strains resulted from an increased rate in the intron regions, as the flanking regions showed no different instability. Furthermore, H_2_O_2_ at the dose used in this study was not able to further increase the global GCR instability in *pol32∆* strains, although it should be noted that the pcm-ble flanking region of intron 6-containing strain and the his-can flanking region of intron 3-containing strain appeared to be significantly more susceptible to H_2_O_2_ treatment. It is possible that the H_2_O_2_-induced GCR formation in *pol32∆* mutants may be either too rare or leading to lethal events not detected by the GCR assay.

The absence of Rrm3 had no effect on global GCR rate of intron 3-containing strain but reduced the spontaneous global GCR rate of the intron 6-containing strain, thus abolishing the significant difference in GCR rates between two strains (Table 2). Detailed analysis revealed the absence of significant difference in GCR rates regarding all three sub-regions. This suggests that the helicase Rrm3 is at the origin of the difference for the differential spontaneous stability of the introns 6 and 3. Furthermore, H_2_O_2_ increased the GCR formation in both strains without distinction according to the nature of the introns and thus acts as an amplifier of the spontaneous susceptibility to break (Table 2).

The absence of Elg1 resulted in 4- to12-fold increase in spontaneous GCR rate in both strains (Table 2). The intron 6-containing strain displayed a higher global GCR rate and higher [his^−^phleo^R^] GCR rate than those of intron 3-containing strain. *elg1∆* mutants with intron 6 or intron 3 context were susceptible to H_2_O_2_-induced GCR formation, with the most instable region being found again in intron 6. Thus, Elg1 does not seem to be involved in the sequence-specific instability of the two introns. The high susceptibility of *elg1*∆ cells to H_2_O_2_-induced GCR formation indicates that oxidative damages trigger replicational impediments that necessitate the action of Elg1.

The absence of Mrc1 resulted in a ~10-fold and a less than 2-fold increase in global GCR rate in intron 3- and intron 6-containing strain, respectively, leading to a similar global GCR rates for both strains (Table 2). The GCR rates in the flanking regions of both strains were also similar. This observation held true also after H_2_O_2_ treatments. Thus, in the absence of Mrc1, all the differences regarding GCR formation between two strains are abolished, strongly suggesting that Mrc1 is a component of the machinery that is, like Rrm3, at the origin of the sequence-specific instability between the two PML introns.

### Mapping of GCR breakpoints by PCR analysis

To have a more precise insight into the events occurring during GCR formation, we selected [his^−^phleo^R^] clones and performed a set of PCR in order to determine the positions of DNA breaks. Every selected clone contained the *ble* gene and lost at least part of the *HIS3* gene (Fig 1). Putnam *et al*. have measured the GCR rates as a function of the distance between the *CAN1*-*URA3* cassettes and *PCM1*, the first essential gene in the chromosome V region [19]. From their data, an exponential decrease of the GCR rates as a function of the distance between *PCM1* and *can1* can be drawn, indicating that the probability of DNA breaks is constant in this region. A more precise study by the same authors showed that the frequencies of breaks were higher in the *can1* region and near the *PCM1* region [16]. Thus, in the present study, the intron 3 and 6 were inserted within a region where the probability of break is constant [16, 19].

**Fig 2:**
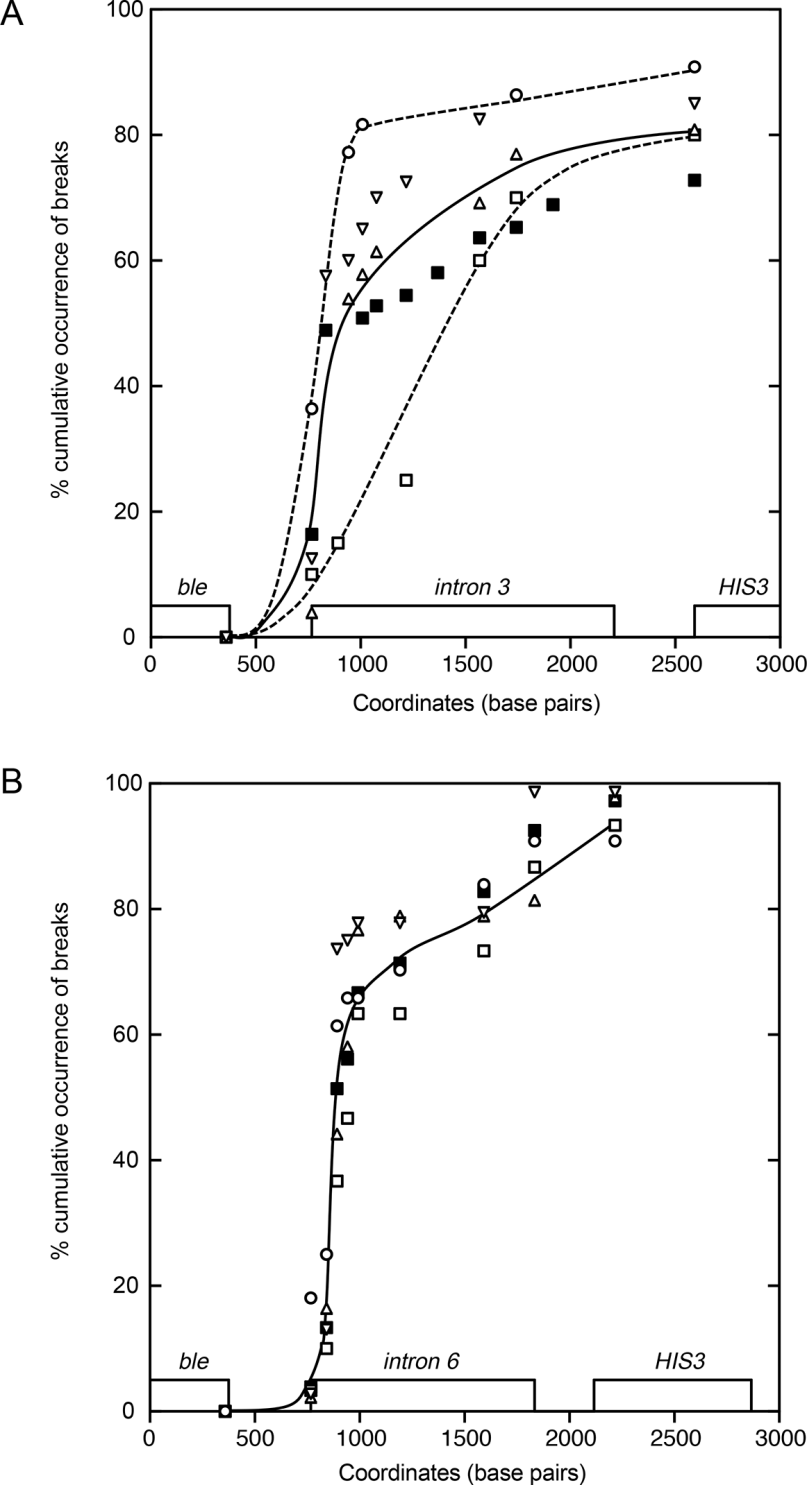
Distribution of cumulative frequency of occurrence of breaks as a function of chromosome V coordinates from the *ble* gene in intron 3-containing strains (A) and intron 6-containing strains (B). Genetic context of strains are indicated as follows: wild-type, closed square; *pol32∆*, open circle; *rrm3∆*, triangle; *elg1∆*, inverse triangle; *mrc1∆*, open square. Curves were drawn using Stineman Function (smooth) of Kaleidagraph software.

Breakpoint positions were mapped by PCR analysis on DNA extracted from the mutants using a set of primer pairs covering the *ble-HIS3* region (Fig 1). By analysis of the primer pairs that did or did not amplify fragments from mutant DNA, breakpoints were mapped to 50–100 bp resolution in the 3’ end of introns 3 and 6. We plotted the cumulative frequencies of occurrence of breaks among [his^−^phleo^R^] clones as a function of the distance from *ble* gene, in the intron 3- and intron 6- containing strains respectively (Fig 2A and 2B). Around 55–60% of the breaks among [his^−^phleo^R^] clones localized within the intron 3. Fig 2A shows that the wild-type, *elg1∆* and *rrm3∆* intron 3-containing strains behaved similarly; the distribution of breaks presents a biphasic response with ∼9–16% of the breaks occurring in the spacer sequence between *ble* and the 3’ end of intron 3, and ∼33% of the breaks in the following 70 bp near the 3’ end of the intron 3 and ∼1.5% of the breaks in the next 60 bp window. The remaining part of the intron 3 seemed to be equally susceptible to breaks as revealed by the monotonous increase of the curve. In *pol32∆* strain there is an increase of breaks (∼35%) in the spacer region between *ble* and the 3’ end of intron 3, and ∼42% of the breaks in the following 70 bp near the 3’ end of the intron 3; thus representing a total of 77% of all the breaks. On the contrary, *mrc1∆* strains presented a more even distribution of the breaks (7% and 10% in the two regions) compared to the other three strains (Fig 2A). In the intron 6 strains, ∼84% of the total breaks of the [his^-^ phleo^R^] clones occurred within the intron 6. The breaks were less equally distributed than that observed in the intron 3 strain. There is no difference between the different mutant strains (Fig 2B). Almost 50% of the breaks occur in a region of 180 bp in the 3’ region of the intron 6. As a whole, it appears that the region near the 3’ end of intron 6 is more susceptible to breaks than the rest of the intron, whereas this susceptibility is somewhat lower in intron 3 except in *pol32∆* strain. Taken together, GCR rate and breakpoint mapping analysis highlight two features that are different between the intron 3 and intron 6: the propensity to generate breaks and the overall sequence specificity.

### Rearrangement breakpoint analysis

**Fig 3:**
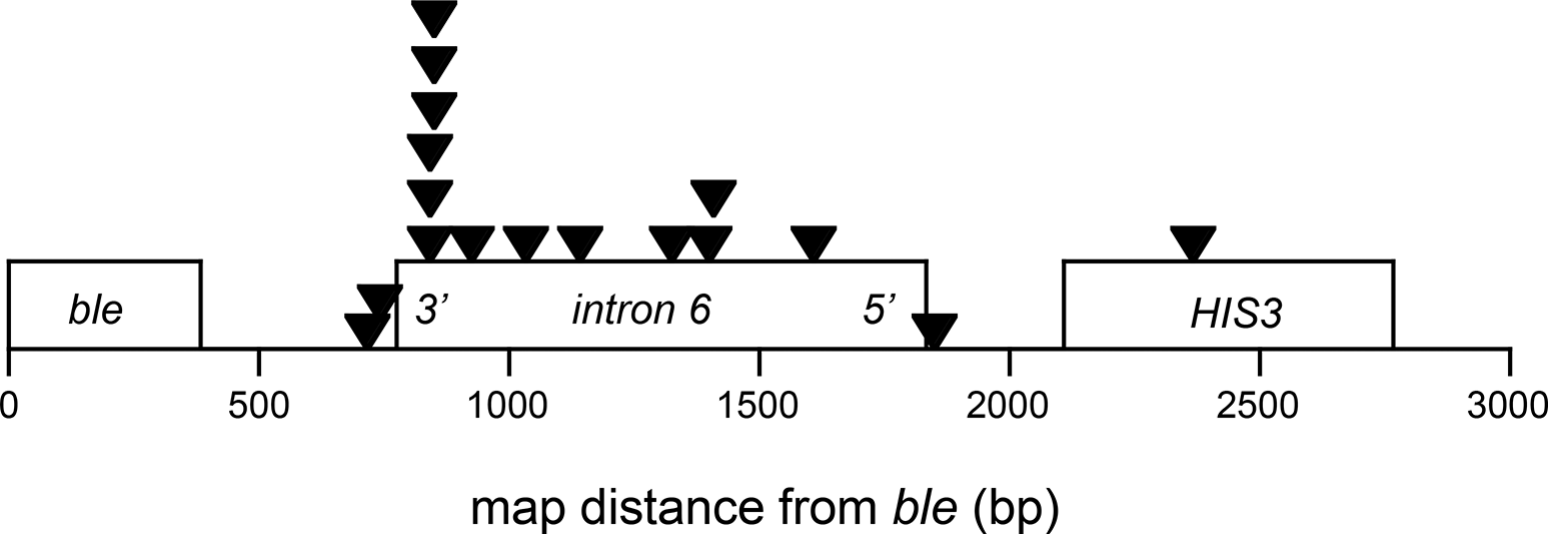
Distribution of intron 6 breakpoints identified by breakpoint mapping and sequence analysis in wild-type intron 6-containing strain. *ble*, intron 6 and *HIS3* are represented by boxes. Black triangles indicate DNA breakpoint positions drawn by Kaleidagraph software.

Finally, in order to determine precisely the breakpoint structure, we analyzed the breakpoint sequences of 17 independent [his^−^phleo^R^] clones obtained from intron 6-containing wild-type strain (combining non-treated and H_2_O_2_-treated strains) using the method described by Kolodner’s laboratory [14]. As expected by using this GCR assay system, the majority of the GCR events (16 out of 17) corresponded to a deletion of an arm of chromosome V combined with the addition of a new telomere (referred to as telomere addition), and one is a complex event with an inversion of surrounding sequences which was not analyzed further. Analysis of breakpoint positions revealed that 6 of 13 events clustered in a 50-bp region near the 3’ end of the intron 6, another 7 events distributed in the remaining part of intron 6, and 4 outside intron 6 (Fig 3). This result further confirms that the region near 3’ end of the intron 6 is the “hotspot” susceptible to DNA breaks. We then compared the sequence homology between intron 6 and intron 3 using EMBOSS Matcher program. Two regions of intron 6, of 45- and 115-nucleotide long, share moderate sequence homology with intron 3, 63% and 60% identity respectively. Among the 13 precisely mapped breakpoints, only one was located within a homologous region. Therefore, the majority of breakpoints of intron 6 occur on the non-homologous region, further underlining the intrinsic genomic instability of intron 6 over intron 3.

## Discussion

In the present study, we used a specific yeast assay to study the molecular mechanisms underlying the instability of the PML gene introns implicated in the t(15;17) translocation. We demonstrate that the intron 3 is a “neutral” sequence for GCR formation as the GCR rate of the intron 3-containing strain is similar to that of wild-type strain without intron (this study) or strains containing a similar window to produce GCR [19]. Intron 6 on the other hand was found to be ∼10 times more instable than intron 3. It is interesting to compare the proportion of bcr1 versus bcr3 in APL patients with the frequency of GCR events affecting intron 6 versus intron 3 in yeast assay. In the present study, 174 independent cultures of intron 6-containing wild-type strain produced 104 GCR events, 72 of them occurred within intron 6, while the same number of cultures of intron 3-containing strain produced 80 GCR events, 38 of them occurred within intron 3, leading to ratio bias of 2/3 for intron 6. In human, about 2/3 of the breakpoints involving PML gene are localized in the intron 6 [3,4]. Therefore, our finding indicating that intron 6 is more susceptible for spontaneous break is consistent with APL clinical data. Thus, the yeast assay is able to mimic the instability of the PML introns implicated in the t(15;17) translocation and allows to conclude that the instability is due to an intrinsic property of the intron sequence in a given genetic context.

**Fig 4:**
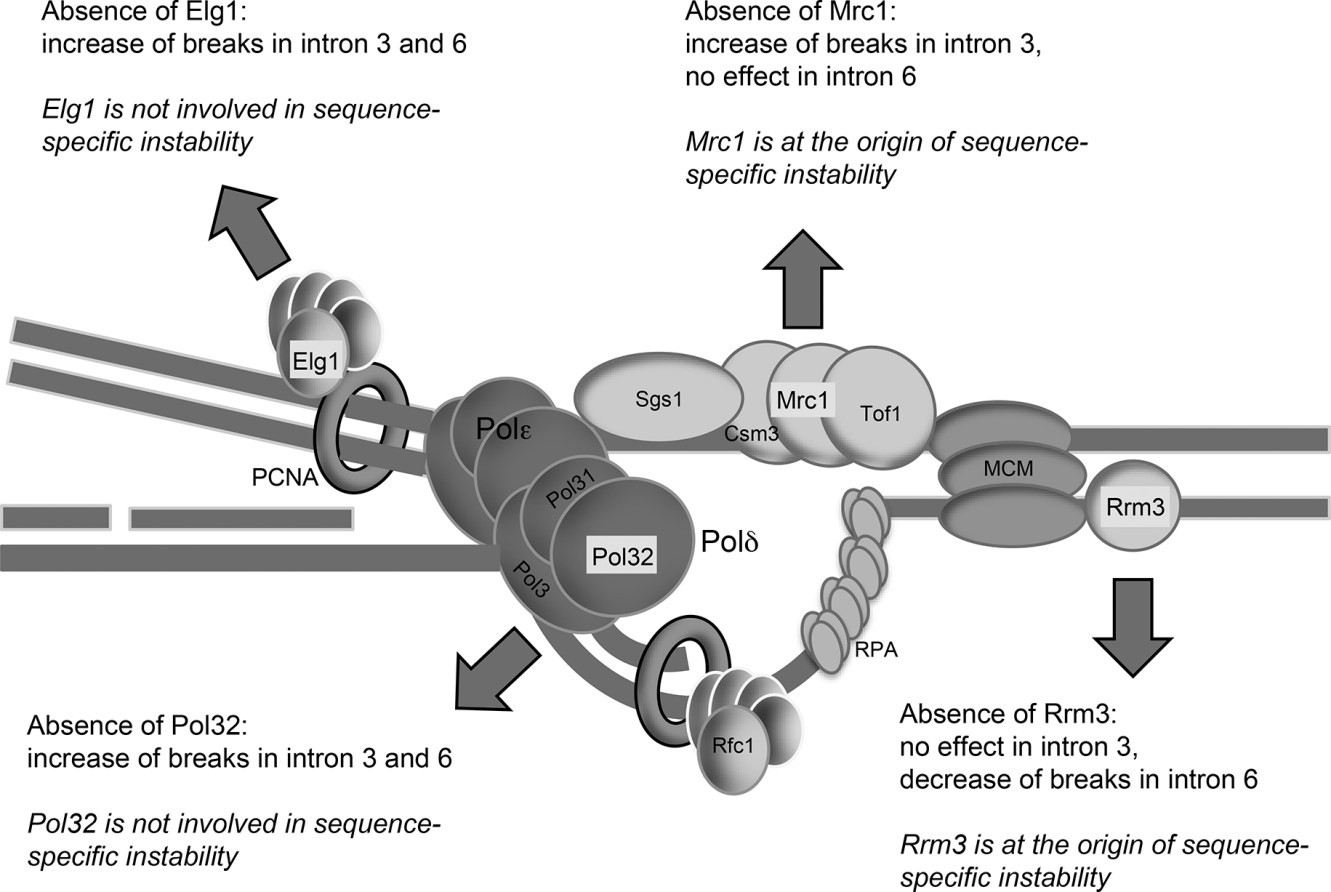
Schematic representation of a replication fork with Pol32, Rrm3, Elg1 and Mrc1 proteins highlighted. The effects of their absence on intron 3 and intron 6 genomic stabilities are indicated.

We studied the impact of 4 candidate genes of interest, *POL32*, *RRM3*, *ELG1* and *MRC1*, all components of DNA replication machinery, in order to gain insights into the genetic and molecular control of the introns’ instability (Fig 4). Analysis of yeast strains invalidated for these genes identified *RRM3* and *MRC1* to be crucial for the instability and the increased susceptibility of intron 6. In *rrm3∆* context, the differences in spontaneous and H_2_O_2_-induced GCR rates between the two introns disappear, indicating that the Rrm3 helicase is at the origin of a sequence-specific susceptibility to form GCR. The absence of Mrc1, the homolog of claspin, that connects the helicase to polymerase ε, abolishes all differences between the two strains, suggesting that Mrc1 also controls the stability in a sequence-dependent manner. Thus, both Rrm3 and Mrc1 known to monitor DNA replication may be involved in the handling of at-risk genomic sequence. On the other hand, although the absence of Pol32 or Elg1 increases global GCR, it does not erase the differential susceptibility of intron 6 over intron 3. These two proteins do not contribute to the sequence-specific susceptibility to form GCR. Since Elg1 is supposed to be involved in the unloading of PCNA behind the replication fork [29], it acts at a late step in the repair of errors introduced during replication, a stage no more affected by the events that are sensitive to sequence context. However, it is important to mention that although we highlight the replication machinery to explain the genetic and molecular control of the instability of intron 6 sequence (Fig 4), the contribution of DNA replication/DNA damage checkpoints should also be taken into consideration. These cellular surveillance mechanisms are intimately linked DNA replication and play crucial roles for maintenance of genomic stability. For example, knockdown of the human DNA helicase RRM3, homolog of yeast Rrm3, enhances phosphorylation of the cell cycle arrest kinase Chk2, indicating activation of the checkpoint via the ATM/Chk2 pathway [34]. By extrapolation in yeast, we could assume that Mec1-mediated checkpoint is very probably activated and this activation is important for maintaining genomic stability and cellular survival in the absence of Rrm3. Indeed, Mec1, yeast homolog of ATM, is essential for the viability of *rrm3∆* cells [26]. Therefore, we cannot exclude the possibility that activation of checkpoint in *rrm3∆* cells, and probably also in *mrc1∆* cells, may contribute to suppressing genomic instability resulted from at-risk genomic sequence.

In patients, the majority of spontaneous APL breakpoints are scattered in intron 6, although not evenly and with no clear-cut hotspot region described. In contrast, analysis of PML breakpoints from a limited number of topoisomerase inhibitors-induced secondary APL points to a “hotspot” which is shown to be the topoisomerase binding site [5–7]. However, the site preference differs between mitoxantrone and epirubicin-related APL. Characterization of 6 cases of epirubicin-related APL showed that 3 were localized within intron 6, with 2 of them positioned within 1 to 2 bp of one another, a statistically significant clustering [6]. The present analysis in yeast assay highlights a higher intron 6 instability and the existence of a fragile region near the 3’ end of the intron 6, relating respectively to the frequency of break and to the distribution of the events (i.e. sequence specificity). One attractive hypothesis is that the breaks leading to GCR are generated via a polar process (replication or resolution of converging forks), which starts (or ends) between *ble* and the introns, moving towards the introns. These sequences are difficult to replicate (or resolve) and the probability of collapse leading to breaks increases as the process runs through the introns. The relationships between the two processes, the frequency of break and the distribution of the events, are complex, since in the analyzed intron 6-containing strains there is little or no difference between the strains regarding sequence-specific break distribution (Fig 2B) whereas there is a 20-fold difference in GCR rates between *rrm3∆* and *elg1∆* strains (Table 2). In contrast, the distributions of the breakpoint events along the intron 3 are not the same according to genetic contexts (Fig 2A). The intron 3 may constitute a weaker replication pausing or resolving site. The probability of collapse is high in the *pol32∆* strain, and lower in the *mrc1∆* strain at the 3’ part of this intron. Mrc1 may form a pausing complex with Tof1, a S-phase checkpoint protein, which may lead to breaks [35]. In the absence of Mrc1, no pausing complex is formed, explaining a more even stalling of replication as replication proceeds into the intron 3.

Other causes for the differential stability of the two human introns have been explored: 1) Presence of G-quadruplexes. A great number of human introns possesses G-quadruplex motifs at the 5’ end of introns [36]. G-quadruplexes are thought to be formed during replication and constitute obstacles to polymerases. G-quadruplexes are prone to GCR induction in a *pif1 rrm3* deficient context [37]. Using QGSR Mapper, we identified 4 sequences susceptible to form G-quadruplexes located at the beginning (5’) of the intron 6 and not near the 3’ end of the intron (the most unstable region with regard to GCR). In intron 3, the 12 putative regions susceptible to form G-quadruplexes are evenly distributed. Therefore, G-quadruplexes are unlikely to be the origin of the differential stability of the two introns in yeast. 2) GC content. Intron 3 has a GC content of 50.7% and intron 6 of 49.9%. Using MeltSim [38], we found that the 3’ part of intron 6 has a higher Tm compared to that of intron 3, reflecting a higher local GC content that may indeed influence the stability of intron 6. 3) Common fragile sites. Common fragile sites corresponding to region with low-density replication origins are regions of human chromosomes prone to breakage [39]. Human common fragile sites are also unstable when inserted in yeast [40]. However, the intron 3 and intron 6 sequences are not related to these structures. Furthermore, in our yeast assay, since the two introns are both inserted in the same chromosomal region, it is very unlikely that the difference of stability of the introns is linked to the yeast replication origins. Symmetrically, this may also be unlikely for implicating termination of replicons. 4) Preferential sites of fixation of topoisomerase II. The origin of some therapy-induced secondary APL is attributed to a specific susceptibility of a short sequence in the intron 6 to fix topoisomerase II, leading to double-strand breaks after epirubicin or mitoxantrone treatments [5,7]. In the yeast assay, the most “fragile” part of the intron 6 is located at the 3’ end of the sequence, some 250 bp away from the putative preferential sites of fixation for topoisomerase II. However, we cannot exclude that topoisomerase II binding-sequences may play a role in the instability of the strain studied, if the processes are different in yeast compared to human cells.

Mutational genome expression profile or SNP studies of APL patients are scarce but, to our knowledge, have not reported abnormalities in genes coding for DNA replication/repair proteins. Spurred by the results of this study, we performed a preliminary Gene Expression Profile (GEP) study in 12 APL patient samples with either bcr1 or bcr3 breakpoint and analyzed 48,803 gene transcripts and gene ontology pathways (data not shown). Focusing the analysis on genes involved in DNA replication, only terminal deoxynucleotidyl transferase (TdT) was differentially expressed between bcr1 and bcr3 APL, TdT being overexpressed in the bcr3 APL. TdT, as other members of the X family of polymerase (pol λ and pol μ), contains breast cancer susceptibility protein BRCA1 C-terminal (BRCT) domain in their N-termini to mediate protein/protein and protein/DNA interactions in DNA repair and cell cycle checkpoint pathways [41]. Furthermore, TdT can interact with other replicative proteins such as PCNA that function to coordinate polymerase activity during replication, repair, and recombination. It has to be determined whether this differential expression pattern has an impact on the specificity and susceptibility of intron 3 and 6 breaks.

In conclusion, the present study using yeast assay shows that the different susceptibility to produce DNA breaks in intron 6 versus intron 3 of human PML gene is likely linked to an intrinsic property of the sequence. To the best of our knowledge, this study is the first attempt to analyze the stability of short human non-repetitive sequences in yeast. We could exclude the determinant role of some features such as G-quadruplexes, while other more subtle differences such as Tm in some regions in the sequences of the two introns could be the origin of this different susceptibility to produce DNA breaks. Our data further indicate that an heterogonous system such as the yeast GCR assay represents an alternative approach to study in details the properties of single unique non-repetitive human sequence in a genetically controlled situation.

## Supporting Information

S1 Table Primers used for strain construction. (DOCX)
