# Supplementary material for: Yeast Assay Highlights the Intrinsic Genomic Instability of Human PML Intron 6 over Intron 3 and the Role of Replication Fork Proteins
Source: PLoS One. 2015 Jun 8;10(6):e0129222. doi: 10.1371/journal.pone.0129222 (PMC4460018; doi:10.1371/journal.pone.0129222)
Supplement: S1 Table — (DOCX) [file pone.0129222.s001.docx]

S1 Table. Primers used for strain construction

| Name | Sequence |
| --- | --- |
| 663 | AATATTACAACTTATTTCCGTAAATAAAGATAGTACACACGAATCCAAACGTTTATATAGgagcttggtgagcgctagga |
| 664 | AGAAGGGTGTGAAACCACCTCTACCAAACACACCAAGAGATGAACCTAAATCAAATTTTCgcatatgatccgtcgagttc |
| 665 | CCC**AAGCTT**GGGgtaagcacgcacgccaccttcc |
| 666 | GA**AGATCT**TTCctgtagaacacaaagaaggtca |
| 667 | GA**AGATCT**TTCctgaaacggggaaggggagat |
| 668 | CCC**AAGCTT**GGGgtagggaggtgggtagggcag |
| 669 | GAAAATTTGATTTAGGTTCATCTCTTGGTGTGTTTGGTAGAGGTGGTTTCACACCCTTCTgtaagcacgcacgccaccttcc |
| 670 | CAAACTAGTGGTTAATAAAAACAAAGTATGTAAAGAATACTCAGTTATTCATTAGAAGGCttcgacactggatggcggcg |
| 671 | GAAAATTTGATTTAGGTTCATCTCTTGGTGTGTTTGGTAGAGGTGGTTTCACACCCTTCTgtagggaggtgggtagggcag |

For 663 and 664 primers, upper cases refer to *NPR2* regions, lower cases to *HIS3* gene in pRS303; For 665 and 666, upper bold cases refer to HindIII and BglII cloning site respectively, lower cases to intron 3 sequence; For 667 and 668, upper bold cases refer to BglII and HindIII cloning site respectively, lower cases refer to intron 6 sequence; For 669 upper cases refer to *CIN8* region, lower cases to intron 3; 670 upper cases refer to *CIN8* region, lower cases to *ble*; 671 upper cases refer to *CIN8* region, lower cases to intron 6.
